# Relevance maps: A weakly supervised segmentation method for 3D brain tumours in MRIs

**DOI:** 10.3389/fradi.2022.1061402

**Published:** 2022-12-21

**Authors:** Sajith Rajapaksa, Farzad Khalvati

**Affiliations:** ^1^ Neurosciences and Mental Health, The Hospital for Sick Children, Toronto, ON, Canada; ^2^ Department of Diagnostic Imaging, The Hospital for Sick Children, Toronto, ON, Canada; ^3^ Institute of Medical Science, University of Toronto, Toronto, ON, Canada; ^4^ Department of Medical Imaging, University of Toronto, Toronto, ON, Canada; ^5^ Department of Mechanical and Industrial Engineering, University of Toronto, Toronto, ON, Canada; ^6^ Department of Computer Science, University of Toronto, Toronto, ON, Canada; ^7^ Vector Institute, Toronto, ON, Canada

**Keywords:** weakly supervised, superpixels, MRI, brain tumours, segmentation, explainability, CNN - convolutional neural network

## Abstract

With the increased reliance on medical imaging, Deep convolutional neural networks (CNNs) have become an essential tool in the medical imaging-based computer-aided diagnostic pipelines. However, training accurate and reliable classification models often require large fine-grained annotated datasets. To alleviate this, weakly-supervised methods can be used to obtain local information such as region of interest from global labels. This work proposes a weakly-supervised pipeline to extract Relevance Maps of medical images from pre-trained 3D classification models using localized perturbations. The extracted Relevance Map describes a given region’s importance to the classification model and produces the segmentation for the region. Furthermore, we propose a novel optimal perturbation generation method that exploits 3D superpixels to find the most relevant area for a given classification using U-net architecture. This model is trained with perturbation loss, which maximizes the difference between unperturbed and perturbed predictions. We validated the effectiveness of our methodology by applying it to the segmentation of Glioma brain tumours in MRI scans using only classification labels for glioma type. The proposed method outperforms existing methods in both Dice Similarity Coefficient for segmentation and resolution for visualizations.

## 1. Introduction

With the increased usage of medical imaging such as Magnetic Resonance Imaging (MRI) in diagnostic procedures, demand for deep learning-based computer-aided diagnosis systems has also expanded to alleviate the pressure from radiologists. These systems have shown great success and can fast-track examinations of potential malignant cases to provide patients with better care. However, training accurate and reliable deep convolutional neural networks (CNNs) requires large fine-grain annotated datasets (e.g., manual tumour annotation). Nevertheless, such datasets are not widely available mainly because manual annotation is prohibitively expensive. This opens the opportunity to explore weakly-supervised solutions where using weak labels (e.g., global classification), fine-grain information such as region of interest (ROI) segmentation can be obtained. Methods such as attention networks (1) have been proposed as potential solutions. Nie D. et al. proposed an attention-based, semi-supervised deep network for medical image segmentation by incorporating a CNN to produce confidence maps using adversarial learning (1). The authors then used the trained CNN model to incorporate unlabeled images to produce segmentations. However, such solutions require retraining with modified network architectures. This would not be practical in a clinically deployed setting where retraining is not feasible due to limitations such as using models pre-trained on propitiatory data from external institutions. Another group of commonly used methodologies is based on Class Activation Maps (CAM) (2). These methods use the idea of projecting back the weights of the output layer on the last convolutional feature map. However, due to obtaining the feature map from the last convolutional layer, these methods struggle with 3D CNN architectures as they produce low-resolution outputs. This highlights the need for a weakly supervised post-hoc solution to the 3D medical image segmentation problem.

This work proposes the classification Relevance Map, a model-agnostic weakly-supervised segmentation method that generates the ROI for an input image based on a novel optimal perturbation on superpixels. Furthermore, we show that this method can also be used as an effective post-hoc visualization tool for 3D CNN architectures to improve interpretability for clinical usage by generating detailed ROI. Finally, we apply the proposed method to the brain tumour segmentation task. Our main contributions are summarized as follows:

• Relevance Map, a Post-hoc explainability algorithm to generate segmentations using perturbation.
• Optimal Perturbation: a method that generates the most effective perturbation given a segmented region of an image.

## 2. Background

### 2.1. Brain tumours

Brain tumours are a collection of neoplasms that are abnormal tissue developed when cells grow and divide in the brain (3). These neoplasms are also referred to as intracranial neoplasms as they have their own specific biology, prognosis and treatment plans. Brain tumours can be benign (not cancer) or malignant (cancer), and they can have a significant effect on the patient’s quality of life (4, 5). Issues can be both general, such as headache, anorexia, nausea, seizures and insomnia. It can also cause secondary issues due to neurological deterioration, such as personality changes, cognitive deficits and visual field defects (6).

Glioma tumours are the most commonly occurring brain tumour type (5). World Health Organization (WHO) has categorized these tumours into four grades (7). Grade I and II belong to the low grade, and grades III and IV are considered high grades. Grade I tumours are least likely to be malignant and possibly curable through surgery. They are also slow-growing and give long-term survival to the patient. Grade II tumours are relatively slow-growing but may recur as high grade, and they are somewhat infiltrative. Grade III is malignant and infiltrative and may grow to be upgraded to grade IV. Finally, grade IV is the most malignant and aggressive. Each type can then be broken down into more subtypes depending on other factors. These categorizations help to determine the proper care for the patient.

#### 2.1.1. Imaging for tumours and radiology pipeline

As the first step, patients will be assigned a type of imaging. The capabilities of each imaging type vary. However, Magnetic Renascence Imaging (MRI) has become the go-to approach for brain tumour evaluation. In this section, we will briefly introduce a typical workflow for diagnosing a patient with a brain tumour. Once the images are captured, they are sent to a neuroradiologist. Neuroradiologists examine and evaluate the captured images of the spine, head, neck and nervous system. They usually compare different MRI sequences, decide on a tumour catagory and recommend further analysis, such as a biopsy. They also utilize Computer-Aided Diagnostic (CAD) systems. CAD systems allow for more efficient and accurate diagnosis. Modern CADs are responsible for image pre-processing, segmentation, feature extraction and classification. These systems are powered by traditional machine learning and deep learning approaches (8). AI for radiology looks to improve these systems. Segmentation of tumour regions plays a key role in improving these methods. For example, radiomics relies on obtaining features within the tumour. However, the current process of segmentation generation involves a large amount of human expert involvement. As most learning methods require expert annotation, a radiologist would be required to mark the region of interest for a given MRI scan. This takes valuable time away from a radiologist and also limits the data available for training such systems as the process is costly. In this work, we focus on this problem, can we generate ROI segmentations without needing expert annotations for deep learning methods?

#### 2.1.2. Brain tumour classification and segmentation

Classification and segmentation have been key interests in the field of AI for medical imaging. Accurate and fast classification of tumours allows for better care for the patient (9). Segmentation of the tumour’s region of interest (ROI) is often performed before the classification. This allows the model to learn features specifically within the tumour leading to better classifications. Furthermore, once segmented, these ROI regions can be used to accomplish much harder classification tasks such as patient survival predictions or O6-Methylguanine-DNA Methyltransferase (MGMT) promoter methylation status classifiers (10, 11).

In this work, we focus on Glioma tumours. With the introduction of the Multimodal Brain Tumor Segmentation Challenge (BraTs) (12–15), several successful methods have been proposed for the classification of HGGs vs. LGGs (16, 17). However, most of these methods rely on tumour (ROI) segmentation before classifying or pre-identifying a 2D slice with tumour ROI present, requiring fine-grain expert annotations. Rehman A. et al. proposed the use of 3D CNN to segment the tumour, followed by a VGG19 classification model trained on the segmented ROI. This method achieved an accuracy of 98.32%, 96.97%, and 92.67% in BraTs 2015, 2017 and 2018 datasets (18), respectively. Haq E. et al. proposed a 2D CNN classifier on pre-selected slices utilizing a region proposal network (RPN) (19). This method achieved an accuracy of 96.5% on the BraTS 2018 dataset. Some direct 3D CNN-based classification approaches have been proposed to classify tumours using the whole volume, relying only on global labels. Shahzadi I. et al. proposed a CNN-LSTM cascade network to classify 3D brain MRI for LGG and HGG tumours (20). On BraTs 2015 dataset, they achieved a classification accuracy of 84%. Mzoughi H. et al. proposed using a Deep CNN model with a pre-processing pipeline and achieved a classification accuracy of 96.5% on the BraTs 2018 dataset (21). Most direct 3D volume classifications lag on performance achieved by pre-segmentation methods and are limited to simple classifications given missing ROI. This highlights the critical role pre-segmentation plays in classification tasks. This work applies our proposed method to generate ROIs from 3D CNN-based full-volume classification models.

### 2.2. Weakly supervised models

Weak supervision can be defined as a model trained with only a subset of information. They can be broken into two categories, ones that require partial annotations such as seed points or bounding boxes and ones with only global-level global classification labels. Weakly supervised methods have been implemented on various medical imaging-related problems. Gama P. et al. proposed few-shot semantic segmentation with sparse labelled images for chest X-ray lung segmentation (22). Roth H. et al. proposed an inexact supervision-based method for segmentation of 3D CT scans of abdominal organs by first generating a point of interest with a random walker algorithm (23). This method achieved comparable DSC scores against a fully supervised deep-learning approach. On the spleen segmentation task, the best performing weakly supervised method achieved a 0.948 DSC score vs 0.958 DSC score for the fully supervised. This motivates us to achieve comparable results with fully supervised methods. Weakly supervised methods have also been used in many tasks within deep learning. Kolenikov et al. proposed seed expansion as a deep learning technique for image segmentation (24). This method reduced the need for extensive human labelling by using only seed labels. It achieved an average detection precision of 0.47 compared to the 0.50 fully supervised method. Methods that utilize only global level labels commonly use Class Activation Map (CAM) based approaches (25, 26). However the methods are mainly focused on 2D images.

### 2.3. Occlusion analysis

Occlusion analysis refers to where input is occluded to observe a change in the model’s output. These changes can then be used to construct heatmaps to identify regions of interest. Similarly, in deep learning, perturbation refers to manipulating inputs to extract information from the model or mislead them. Eykholt et al. highlighted the effectiveness of perturbations by manipulating physical objects that are commonly used for guiding self-driving cars (27). For example, the authors showed adding black and white stickers to road stop signs would lead the model to misclassify them as speed limit signs. Therefore, the model has learned black and white stripes are associated with speed limit signs. Similarly, Szegedy C. et al. showed that generalized deep learning models could be misled by adding hardly any visual perturbation, such as noise, to the input image (28). These works highlight using perturbation as a method to extract information. The local Interpretable Model-Agnostic Explanations (LIME) technique was proposed to use localized perturbations as a model-agnostic method for extracting regions of interest to the classification model (29). It attempts to increase model interpretability by evaluating the model’s confidence when images are perturbed. In this method, For n iterations, a set of superpixels would be selected and perturbed to assess its effect. Each superpixel group would then be assigned a score depending on the classification confidence change. Using these scores, a secondary classification model is trained to determine if the selected superpixel significantly affects the classification. This method was applied to a wide array of applications given its model-agnostic approach (30–32). Most applications are evaluated using qualitative analysis on its ability to highlight ROI. Randomized Input Sampling for Explanation (RISE) further improved this work by introducing randomized, global masking to calculate region relevancy to a classification by removing the localized superpixel borders (33). RISE would generate n random masks to perturb the model and assign each mask with a score depending on the change in classification confidence. Once all n masks are summed together, the region of interest has the highest score. Both of these works showed high adaptability as they are model-agnostic and can be easily used on any pre-trained model. Yu L. et al. utilized RISE to generate explanations for Alzheimer’s disease diagnosis model (34).

### 2.4. Superpixels

Superpixels are groupings of perceptually similar pixels to create a meaningful image with fewer primitive elements for processing. The term coined by Ren and Malik in Learning a Classification Model for Segmentation set out to solve the problem that as pixels are not natural entities, they are meaningless as representations of images (35). Superpixels have been used to segment medical images as they capture similar subregions in images such as tumours (36, 37). Simple Linear Iterative Clustering (SLIC) (38) propose generating superpixels by clustering pixels based on their colour similarity and proximity in the image. Using a five-dimensional space consisting of colour, either LAB or RGB and the x and y coordinates, the authors propose using a distance measure that accounts for the difference within the 5D space. The proposed algorithm would then initialize the cluster centers in the given image and repeat until all clusters have achieved the best match pixels according to the distance measure. This method achieved a complexity of O(N) where N is the number of pixels. This method has been widely used for its efficient and accurate representation of the groupings.

## 3. Methods and material

### 3.1. Dataset

The Multimodal Brain Tumor Segmentation Challenge 2020 dataset was used as our dataset (12–15). 3D volumes of T1-weighted (T1w), T1-weighted with contrast enhancement (T1wCE), T2-weighted (T2w) and FLAIR sequences were available for each patient, along with tumour segmentation and type (HGG or LGG) (Figure 3.1). Three hundred sixty-seven scans were available in the dataset, with 263 being HGG class and the remaining being LGG. Only 190 3D scans (training: 133, validation: 19, test: 38) were randomly selected to maintain a 60% HGG and 40% LGG tumour ratio to balance the training classes. Each sequence was independently normalized using min-max normalization, and the center cropped to 128×128×128 volumes.

### 3.2. Method

**Figure 1:**
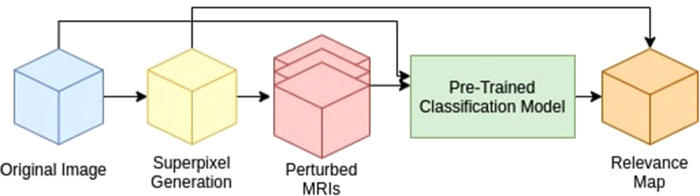
Proposed method pipeline. We divide our method into three main components: 1. Superpixel Generation, 2. Perturbation, and 3. Relevance Map generation.

As shown in Figure 1, our method is comprised of three main components:

1. Generate a superpixel map for a given input.
2. Iterate through the superpixels to create a perturbed input to the pre-trained CNN model.
3. Measure the trained CNN probability difference for each perturbed input compared to that of the original image to generate the Relevance Map.

We have selected a 3D Resnet 50 (39) model as our baseline classification architecture to evaluate the proposed method. We selected Resnet 50 as it represents a commonly used classification model in our field. The model was trained on all four sequences (T1w, T1wCE, T2w, FLAIR) with a shape of 4×128×128×128 pixels as input with a learning rate of 0.01 and the Adam optimizer (40) for 100 epochs using binary cross-entropy as the loss function. The trained model at epoch 81 was selected for evaluation as it achieved the highest validation AUC with 0.86. The model achieved an area under the ROC curve of 0.83 on the test set.

### 3.3. Superpixel segmentation

**Figure 2:**
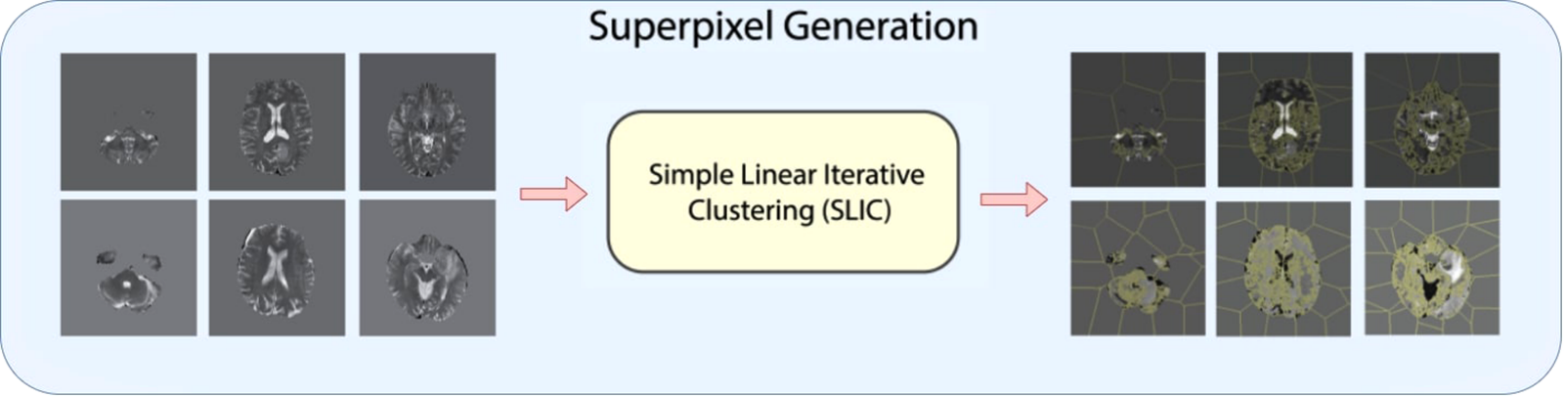
Superpixel generation step. 3D MRI volumes are processed using SLIC algorithm to generate 3D superpixel groupings.

**Figure 3:**
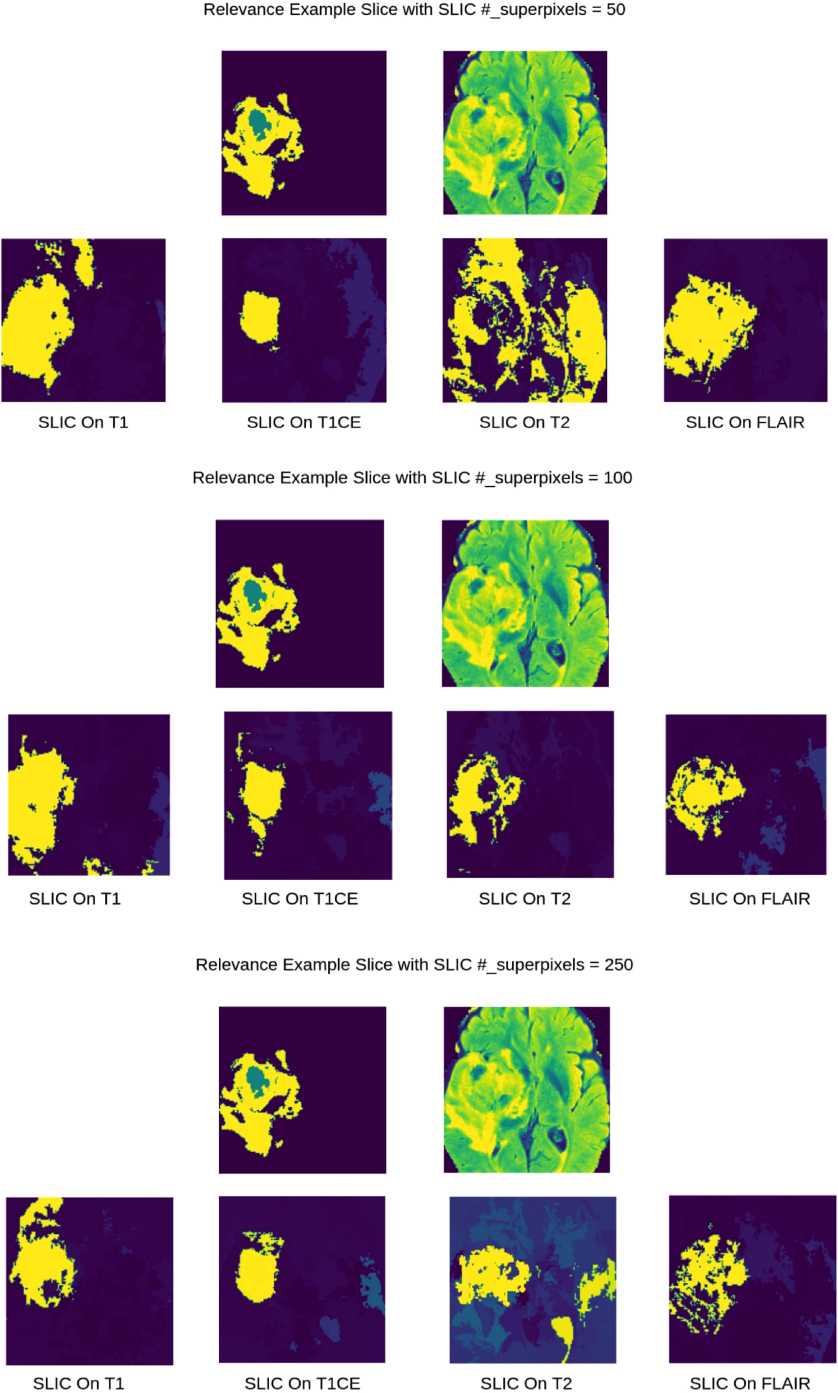
2D visualization of Relevance Maps generated with 50, 100, 250 as the starting number of superpixels on their respective MRI modality. This highlights the difference in superpixel generation parameters can affect the Relevance Maps.

As shown in Figure 2, for 3D superpixel generation, Simple Linear Iterative Clustering (SLIC) was used (38, 41). We conducted a grid search using validation data as part of the experimentation to determine the best parameters for sequence type for superpixel generation and the number of superpixels. Figure 3 shows that the number of superpixels and the sequence type plays a critical role in selecting a region of interest. The best set of parameters was selected based on the Sørensen–Dice coefficient score achieved on tumour segmentation with the best superpixel grouping, where we selected the best performing threshold level. Our grid search for superpixel parameters conducted on the validation set showed that 100 superpixels generated on the T2w sequence yielded the best results.

### 3.4. Perturbation

The perturbation of input is applied to the superpixel map generated in the first step. As a baseline, we first introduce three naive perturbation methods:

1. Blank perturbation, turn off all pixels in a given superpixel by replacing its intensity value with zero.
2. Max perturbation sets a superpixel’s value to the max value of the given local region.
3. Min perturbation sets the superpixel’s value to the min of the local region.

#### 3.4.1. Optimal perturbation

We define optimal perturbation as the most effective perturbation for a given region on the trained model. This is measured by the change in the prediction probability of the pre-trained model’s classification output. In other words, the higher the change in the prediction probability of the pre-trained model’s classification output, the more influential the perturbation algorithm is. To determine the optimal perturbation for a given 3D superpixel, we propose training a 3D U-net model (42) with the individual 3D superpixels as the input and a perturbed mask for that superpixel as the output by optimizing the difference between perturbed and the non-perturbed classification.

**Figure 4:**
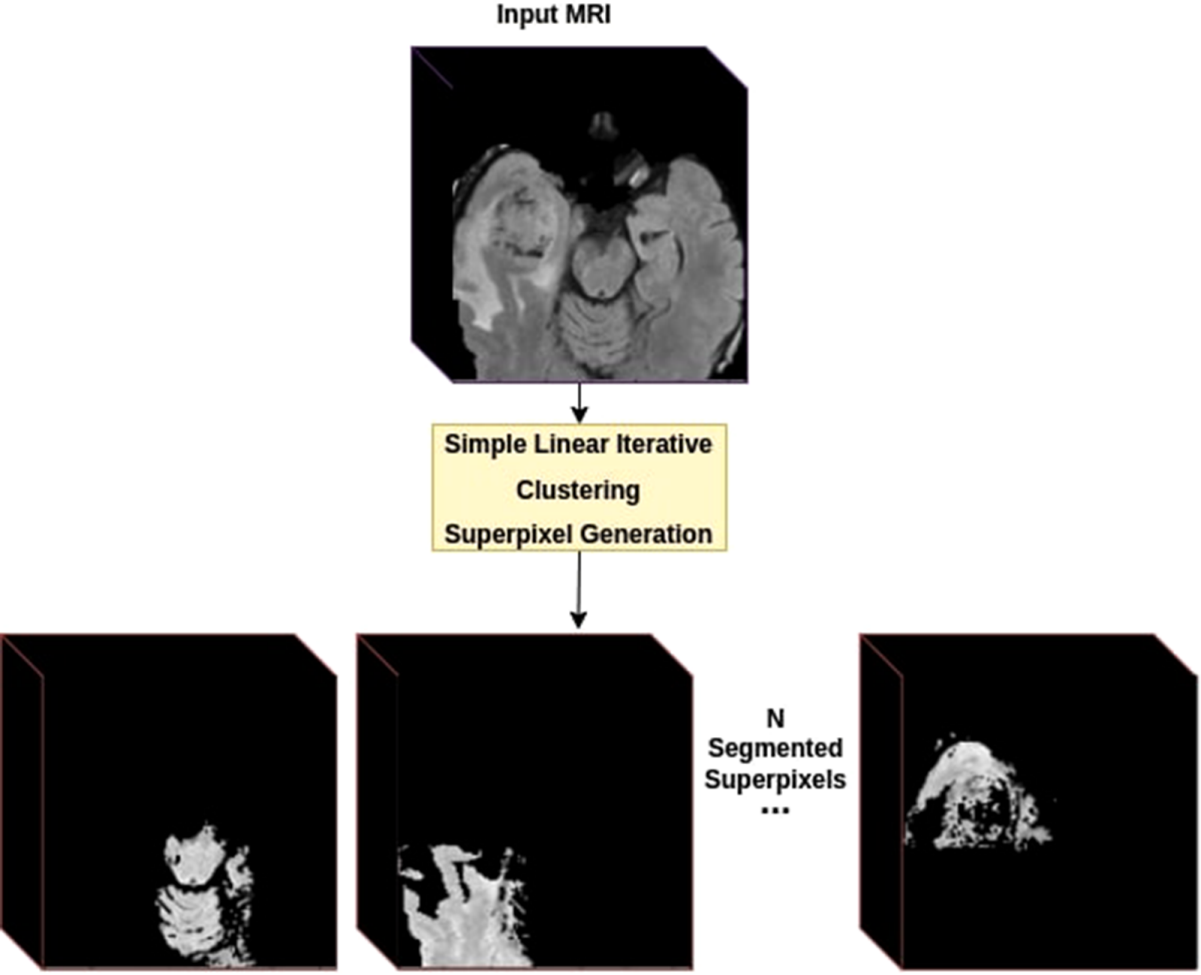
Dataset creation using superpixels. For each provided MRI volume, we generate a superpixel segmentation map using the SLIC algorithm. Each Superpixel is then separated into its own volume as a new training sample.

First, the dataset was generated using SLIC parameters deemed best using the naive perturbation grid search on the training set. Then, all superpixels generation was done on T2w sequence with the number of initial superpixels set to 100. For experimentations, we used six randomly selected MRI samples, three from LGG class and three from HGG class, to generate training samples with the parameters mentioned above. After SLIC iterations, this resulted in a total of 443 samples in the training set as SLIC combines superpixels with too much similarity. Each sample was a 128×128×128×4 volume only containing the selected superpixel (Figure 4).

Then we introduce perturbation loss, which maximizes the difference between the classification probability (generated by the pre-trained ResNet) of the perturbed image and the original image. For each batch, the loss would be calculated by applying (multiplying) the generated perturbations mask onto the original MRI and generating a classification using the pre-trained ResNet. Then we calculate the difference between the perturbed and non-perturbed predictions to determine the effectiveness of that generated perturbation.

$$L_{\mathrm{perturbation}}=\frac{1}{n}\sum_{i=1}^{n}\frac{1}{abs(y_{np_{i}}-y_{\;p_{i}})}$$

Where p is perturbed, np is non_perturbed, y is the classification prediction, and n is the number of samples in a batch.

**Figure 5:**
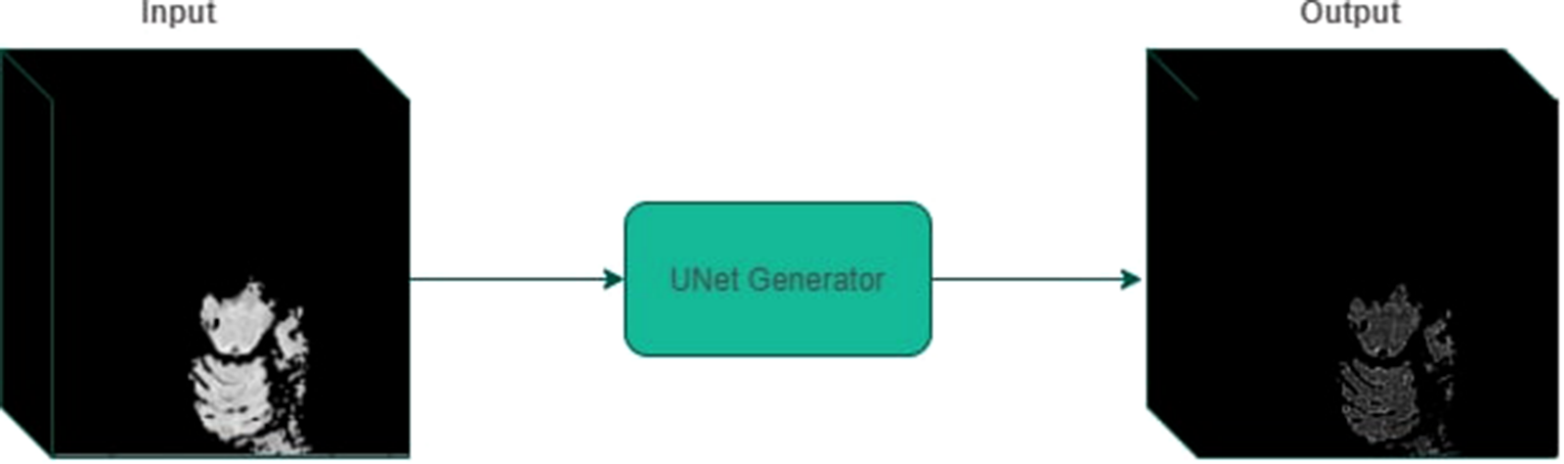
An example of perturbed mask generation using UNet generator. Given a 3D volume containing a segmented superpixel, the generator model outputs a mask that can be applied to the original MRI volume.

Finally, we trained a 3D U-net model on the generated dataset on the Adam optimizer using perturbation loss with a learning rate of 0.01 for 25 epochs. The last epoch was selected to carry out the experimentation. Figure 5 displays an example input/output to the optimal perturbation model.

### 3.5. Relevance maps

**Figure 6:**
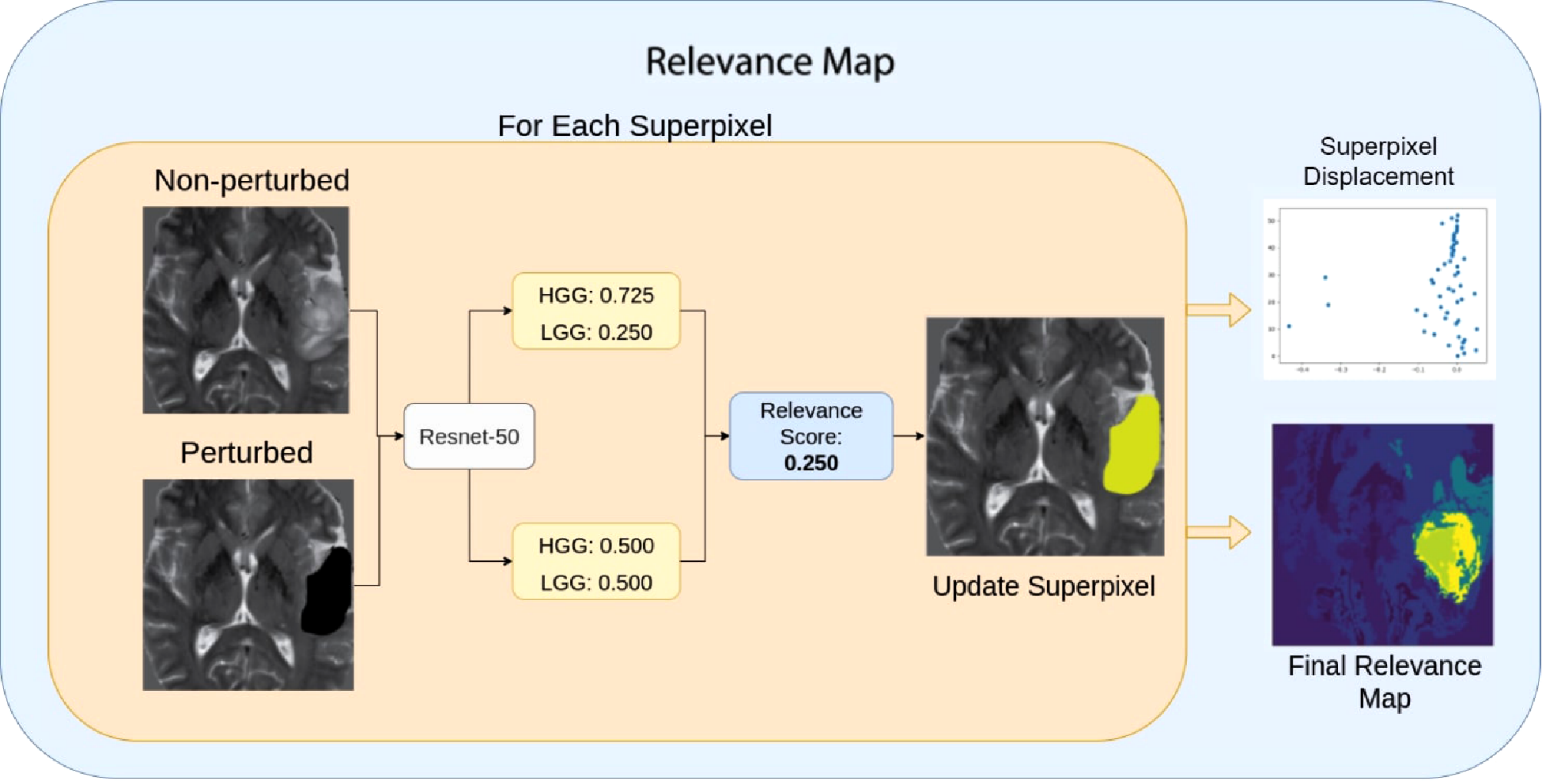
Relevance Map scoring pipeline. For a given superpixel, we compute the difference between the perturbed classification score and the non-perturbed classification score. Then we assign the superpixel with the difference in the score as the region’s importance. Higher scores indicate the most important region.

Finally, we define Relevance Maps as a superpixel segmentation map of the image, each with an associated score on its importance to the predicted classification. Relevance maps were generated by assigning each superpixel a score on its ability to change the model’s confidence on classification by calculating the absolute difference between the pre-trained model’s classification probabilities for perturbed and non-perturbed images. Relevance maps were then normalized between 0 and 100, where regions with 0 scores did not affect the classification, and those with 100 had the highest impact on the classification probability. Figure 6 and Algorithm 1 display visualization and the pseudo-code for the Relevance Map generation. We can then use the ranking system to generate a segmentation by only selecting a given rank. For example, we can combine superpixels associated with ranks one and two to generate the ROI segmentation.

### 3.6. Evaluation

We computed the average Dice similarity coefficient (DSC) against the expert annotations of the brain tumours for all quantitative evaluations. DSC score of 1 indicates a perfect match between the predicted and expert ROI and a DSC score of 0 indicates no overlap.

$$\mathrm{DSC}=\frac{2|X\cap Y|}{\left|X|+|Y\right|}$$

Where |X| represents the predicted set of pixels, and |Y| represents the expert annotated set of pixels.

We conducted the following evaluations:

1. *Evaluation with Best Superpixel Grouping*: First, we evaluate the produced Relevance Maps by generating segmentation with the best superpixel groupings. We iterate through the superpixels from highest ranked to lowest to find the best grouping for a given image when compared against the expert annotation. This evaluation displays the highest achievable DSC by removing the clustering as a bottleneck, as the focus of this work is not on superpixel clustering but on highlighting the ability of ROI extraction.
2. *Evaluation with Ranked Relevance*: Secondly, we calculate the average DSC for each ranked superpixel, where rank one means that the superpixel with the highest value and had the most effect on the classification of the tumour. The selection of the ranked superpixels is independent of any human expert annotation. These are treated as the produced segmentation masks.
3. *Evaluation on Combined Ranked Superpixels*: We evaluate the effectiveness of combining top-ranked superpixels to generate a better segmentation. For each Relevance Map, we calculated DSC score by treating the combination of top ranks as a single segmentation mask. For example, combing first, second and third-ranked superpixels. This indicates whether the lower-ranked superpixels would contribute to the tumour region. When combined with the top-ranked ones. The selection of the ranked superpixels is independent of any human expert annotation.
4. *Qualitative Evaluation of Faithfulness Against Validation Loss*: To ensure that the Relevance Map is faithful to the trained information, we generated a Relevance Map at each epoch while training and compared it to the validation loss. We expect a faithful method to vary its confidence in the regions, similar to the validation loss. It displays the learned knowledge in the model where a low confidence relevance map will represent high validation loss, as a model with high validation loss is most likely to have learned non-generalizable information. This evaluation will give us the confidence to ensure Relevance Maps are actual representations of the model’s knowledge.
5. *Comparison to Grad-CAM and LIME*: Finally, we compare tumour segmentation achieved with the proposed method to other post-hoc methods. We have chosen Grad-CAM and LIME (29) for their widespread use. For Grad-CAM, we have generated visualization for each test case and interpolated it to 128×128×128 from 6×6×6 to calculate DSC using the best binary threshold for the given image. For LIME, we adapted the algorithm to process 3D inputs and used SLIC (38) as the superpixel generation method. The algorithm was run on the test set with the parameters of hide colour set to zero, similar to our blank perturbation, and the number of samples used for classification training was set to 1,000. We then calculated the DSC of the predicted region for the given class.

## 4. Results

### 4.1. Dice similarity coefficient with best superpixel grouping

Table 1 shows DSCs obtained using the best superpixel grouping on different perturbations and superpixel generation parameters with naive perturbations. We found Blank perturbation (among trivial methods) with 100 superpixels generated on the T2w sequence performed the best in the naive perturbations.

**Table 1:** DSC scores with best superpixel grouping on Relevance Maps with naive perturbations. Blank perturbation with superpixels generated on T2w sequence achieved the highest DSC score.

	Blank			Min			Max		
	50	100	250	50	100	250	50	100	250
T1w	0.24	0.27	0.29	0.13	0.15	0.17	0.09	0.1	0.1
T1wCE	0.27	0.27	0.29	0.17	0.19	0.21	0.1	0.1	0.1
T2w	**0.37**	**0.4**	**0.34**	0.21	0.22	0.24	0.1	0.1	0.1
FLAIR	0.33	0.33	0.32	0.24	0.22	0.22	0.1	0.1	0.1

As shown in Table 2, using the same superpixel parameters (T2W, N=100) as the best-performing trivial perturbation method from the previous study, the Relevance Map generated using our proposed optimal perturbation method achieved a **DSC of 0.45**.

**Table 2:** Average DSC comparison between naive and optimal perturbation using best superpixel groupings.

	Naive perturbation	Optimal perturbation
Average DSC score	0.40	0.45

### 4.2. Dice similarity coefficient with ranked superpixels

For the remaining evaluations, we present the comparison between optimal perturbation and blank perturbation as it achieved the highest DSC among the naive perturbations. Table 3 shows the DSC for ranked superpixels where the blank perturbed superpixels generated on the T2w sequence with 100 superpixels achieved an average DSC of 0.25. The highest-ranking optimally perturbed superpixel achieved an average DCS of 0.31.

**Table 3:** Average DSC achieved on different ranked superpixels compared with ranked by optimal perturbation vs blank perturbation.

Rank	Avg. DSC with optimal perturbation	Avg. DSC with blank perturbation
1	**0.31**	0.25
2	0.18	0.07
3	0.10	0.06

### 4.3. Dice similarity coefficient on combined ranked superpixels

As shown in Table 4, the proposed method outperformed blank perturbation when combining the first two superpixels generating an average DSC of 0.34, while combining superpixels selected by blank perturbations reduced the average DSC to 0.22. The proposed optimal perturbations are on average better at picking secondary superpixels that contain tumour regions.

**Table 4:** Average DSC evaluation on cumulative top ranking superpixels compared with ranking with optimal perturbation vs black perturbation.

Rank	Avg. DSC with optimal perturbation	Avg. DSC with blank perturbation
1	0.31	0.25
1+2	**0.34**	0.22
1+⋯+3	0.32	0.18
1+⋯+4	0.29	0.16
1+⋯+5	0.25	0.16

### 4.4. Qualitative evaluation of faithfulness against validation loss

**Figure 7:**
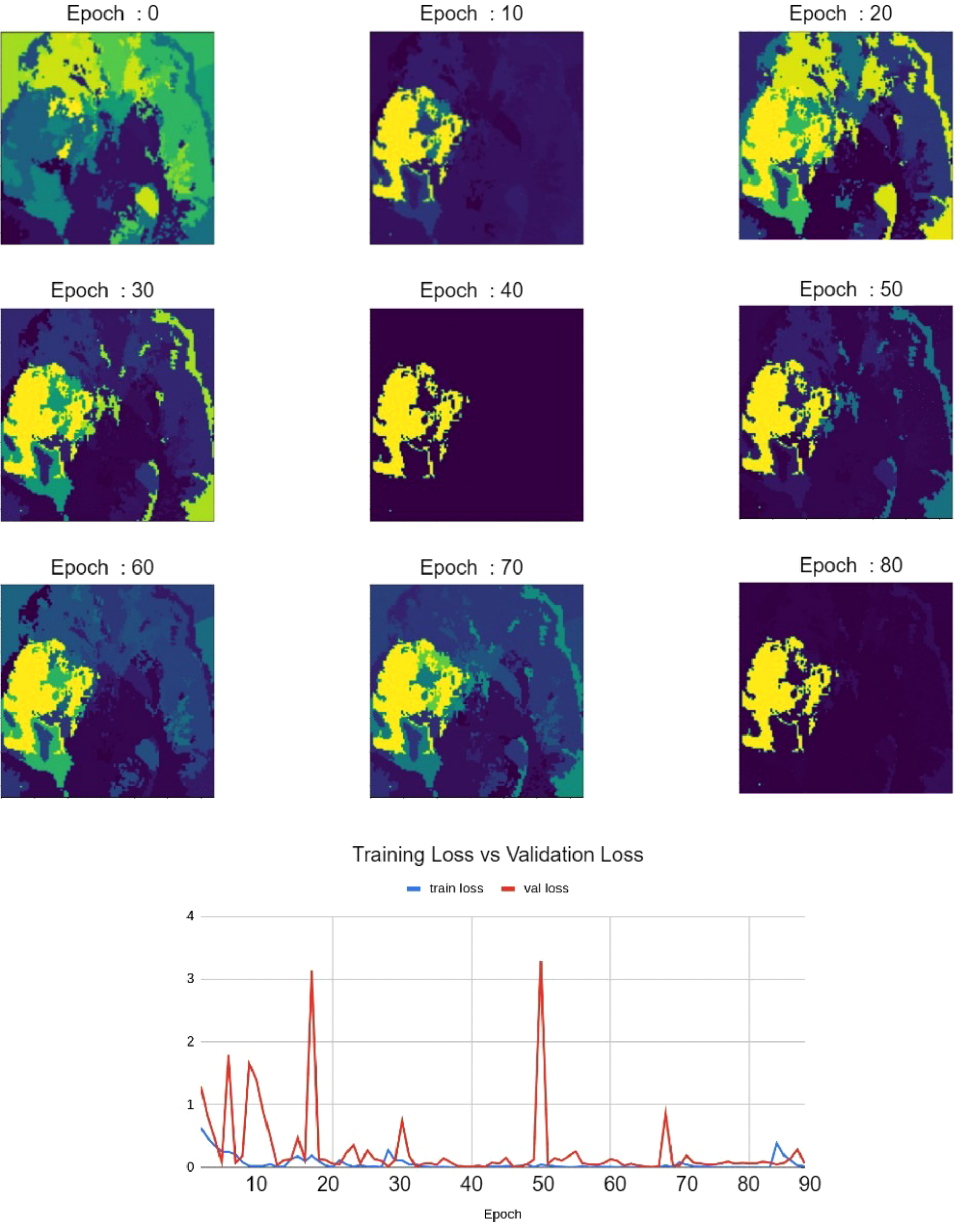
Relevance maps for different training epochs visualizing different levels of focus. This visualizes the improvement of Relevance Maps correlated with the validation loss.

As shown in Figure 7, Relevance Maps follow a similar pattern to the validation loss when visually inspected. High loss values indicate more variability in the relevance map, and low loss values indicate more focused Relevance Maps. This shows a faithful explanation of the learned features of the input image where confidence in the relevance also depends on the validation loss.

### 4.5. Comparison to Grad-CAM and LIME

**Figure 8:**
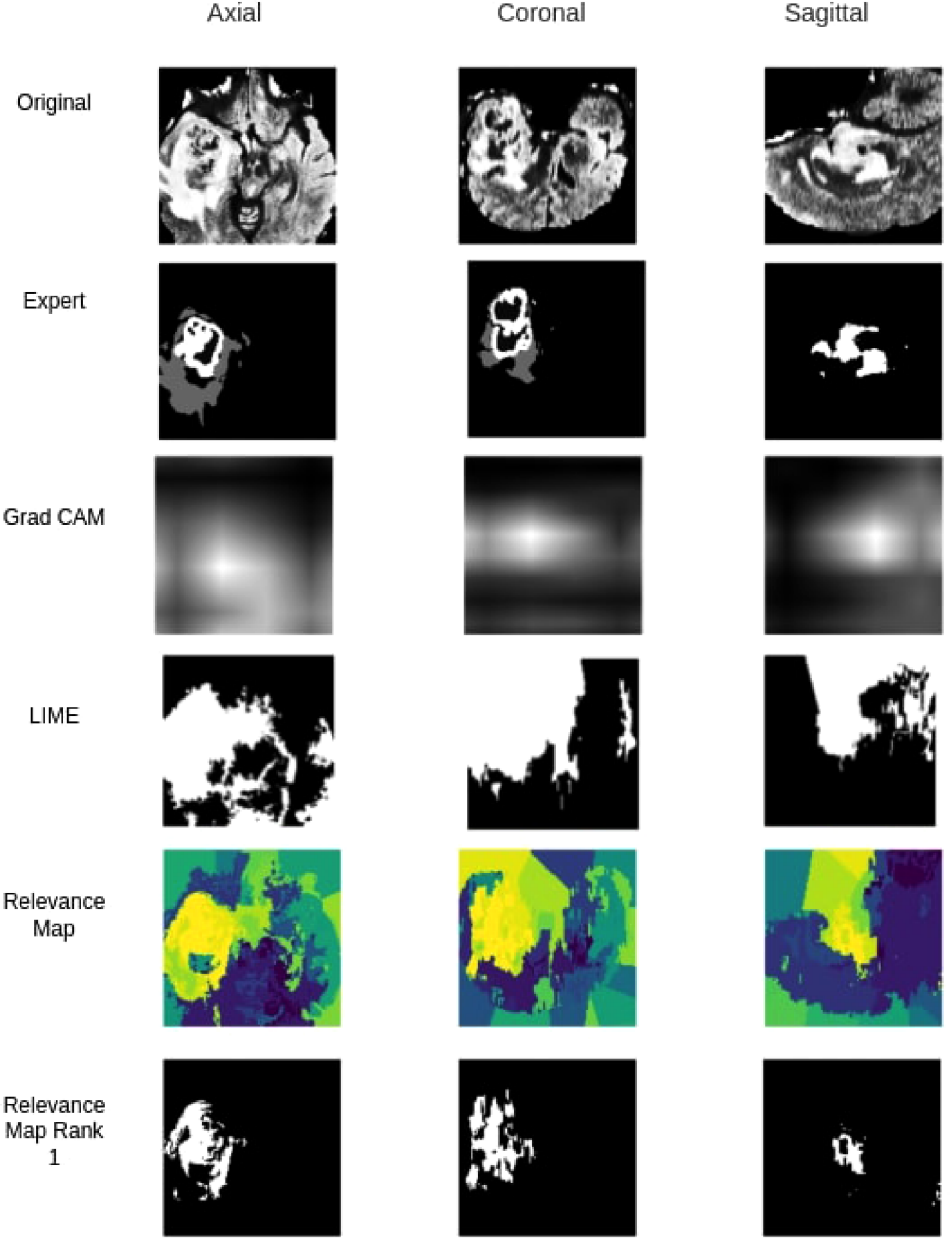
Qualitative segmentation comparison between Grad-CAM,LIME, Relevance Map and Ranked one Superpixel on Relevance Map.

When compared against Grad-CAM and LIME methods, the proposed method outperformed both Grad-CAM and LIME in visualization quality and the ability to localize the tumour. As shown in Table 5, with the best thresholding, Grad-CAM achieved an average DSC of 0.11, while our method achieved 0.45 with the best superpixel grouping. Similarly, LIME achieved an average DSC of 0.06 on the testing set. Figure 8 shows a sample visualization of Grad-CAM, LIME and Relevance Map compared to expert annotations. Our Relevance Map generated a higher resolution and more meaningful visualization of the ROI than the other two methods.

**Table 5:** Average DSC score comparison between Grad-CAM, LIME, Relevance Maps methods using best superpixel groupings.

	LIME (29)	Grad-CAM (43)	Relevance maps (blank perturbation)	Relevance maps (optimal local perturbation)
Average DSC score	0.06	0.11	0.40	**0.45**

**Table:** 

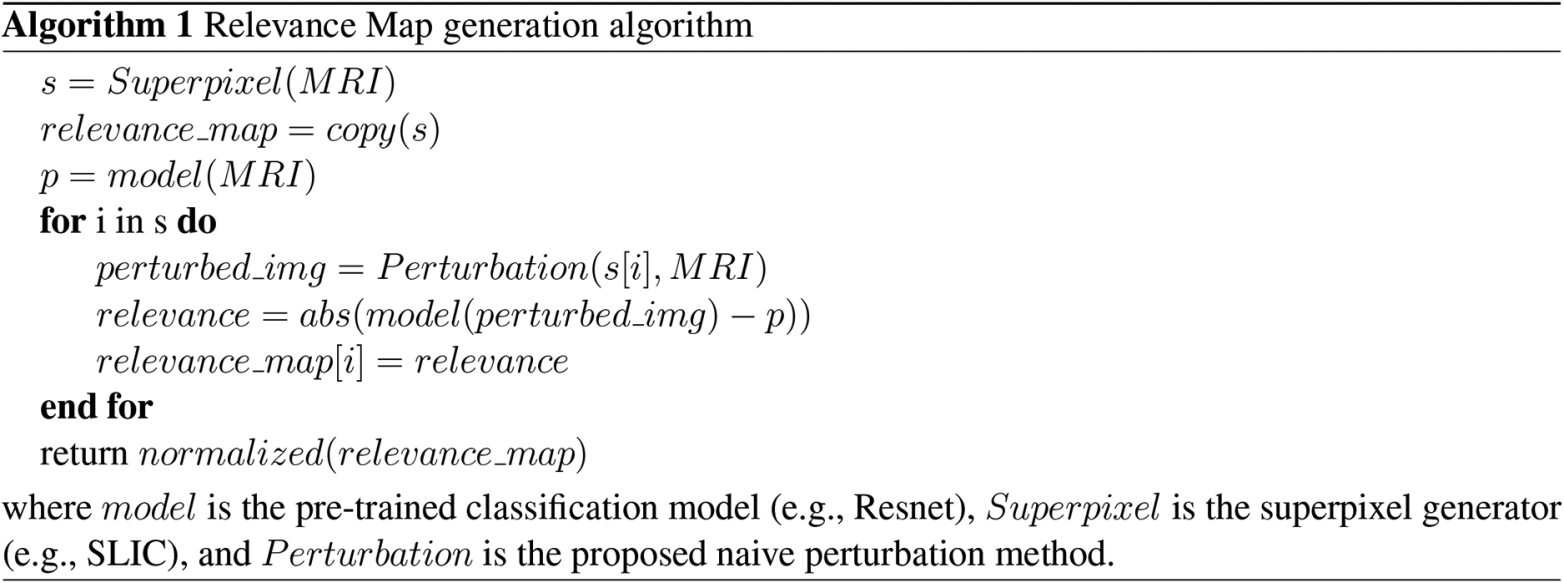

## 5. Discussion

Identifying regions of interest plays a crucial role in model explainability and improvement. We generated high resolution and accurate segmentations using the proposed Relevance Map and optimal perturbation compared with the commonly used methodology. When compared against Grad-CAM, We see a clear improvement in our proposed method in the visual explanation and the DSC score. Furthermore, our results show that even the trivial perturbation methods could outperform Grad-CAM in 3D medial images. We attribute the failure of grad cam to its inability to produce high-resolution output in the 3D space. Since the original output is only a 6×6× volume, it lacks the required information to generate a meaningful segmentation or visualization when interpolated to 128×128×128.

Similarly, our method outperformed LIME, even though LIME follows a similar perturbation approach. It failed to classify an appropriate set of superpixels to the given classification and often would classify background superpixels as contributors to the classification. This behaviour was heavily penalized on the DSC calculation and would be of no benefit to any clinician looking for insight.

Our results also show that the proposed optimal perturbation method can determine more significant superpixels when compared to trivial methods. As shown in Table 2, a Relevance Map generated using optimally perturbation would detect actual tumour regions and rank them as most significant more often than the best performing trivial perturbation. Similarly, Optimal perturbation also selected more tumour regions as the second-ranked. Due to this, as shown in Table 3, we can generate a higher DSC by combing both first and second-ranked superpixels.

Our qualitative evaluation of faithfulness against validation loss highlighted the method’s ability to represent learned knowledge. We showed that as the model validation loss decreased, so is the confidence in our Relevance Map. We see this as of great importance when our method is to be used as an explanation. Especially in a clinical setting, our explanation needs to reflect the knowledge within the classification model. The ability to highlight low confidence provides us with trust that visualization can reflect the learned region of interest.

## 6. Conclusions

In this work, we proposed a novel localized perturbation method to extract the ROI (tumours) from a 3D CNN trained only to classify MRI brain glioma tumours. We also showed that our proposed method of superpixels-based perturbation mask generator (Relevance Map) could also generate visualization maps to significantly improve the interpretability of black-box 3D classification models.

## Data Availability

Publicly available datasets were analyzed in this study. This data can be found here: https://www.med.upenn.edu/cbica/brats2020/data.html.

## References

[1] Nie D Gao Y Wang L Shen D. Asdnet: attention based semi-supervised deep networks for medical image segmentation. In *International Conference on Medical Image Computing and Computer-Assisted Intervention*. Granada, Spain: Springer (2018). p. 370–378.

[2] Zeiler MD Fergus R. Visualizing and understanding convolutional networks [Preprint] (2013). Available at: http://arxiv.org/1311.2901.

[3] McKinney P. Brain tumours: incidence, survival, and aetiology. Journal of Neurology, Neurosurgery & Psychiatry. (2004) 75(suppl 2):ii12–7. 10.1136/jnnp.2004.040741 15146034 PMC1765660

[4] Taphoorn MJ Sizoo EM Bottomley A. Review on quality of life issues in patients with primary brain tumors. Oncologist. (2010) 15(6):618–26. 10.1634/theoncologist.2009-0291 20507891 PMC3227985

[5] DeAngelis LM. Brain tumors. N Engl J Med. (2001) 344(2):114–23. 10.1056/NEJM200101113440207 11150363

[6] Mckee AC Daneshvar DH. The neuropathology of traumatic brain injury. Handb Clin Neurol. (2015) 127:45–66. 10.1016/B978-0-444-52892-6.00004-0 25702209 PMC4694720

[7] Kleihues P Burger PC Scheithauer BW. The new who classification of brain tumours. Brain Pathol. (1993) 3(3):255–68. 10.1111/j.1750-3639.1993.tb00752.x 8293185

[8] Huang Q Zhang F Li X. Machine learning in ultrasound computer-aided diagnostic systems: a survey. Biomed Res Int. (2018) 2018:1–10. Article ID 5137904. 10.1155/2018/5137904 PMC5857346 29687000

[9] Doolittle ND. State of the science in brain tumor classification. In *Seminars in oncology nursing*. Vol. 20. Portland, OR, USA: Elsevier (2004). p. 224–230. 15612598

[10] Yogananda C Shah BR Nalawade S Murugesan G Yu F Pinho M, et al. MRI-based deep-learning method for determining glioma MGMT promoter methylation status. Am J Neuroradiol. (2021) 42(5):845–52. 10.3174/ajnr.A7029 33664111 PMC8115363

[11] Chato L Latifi S. Machine learning and radiomic features to predict overall survival time for glioblastoma patients. J Pers Med. (2021) 11(12):1336. 10.3390/jpm11121336 34945808 PMC8705288

[12] Menze BH Jakab A Bauer S Kalpathy-Cramer J Farahani K Kirby J, et al. The multimodal brain tumor image segmentation benchmark (brats). IEEE Trans Med Imaging. (2014) 34(10):1993–2024. 10.1109/TMI.2014.2377694 25494501 PMC4833122

[13] Bakas S Akbari H Sotiras A Bilello M Rozycki M Kirby JS Freymann JB Farahani K Davatzikos C. Advancing the cancer genome atlas glioma MRI collections with expert segmentation labels, radiomic features. Sci Data. (2017) 4(1):1–13. 10.1038/sdata.2017.117 PMC5685212 28872634

[14] Bakas S Reyes M Jakab A Bauer S Rempfler M Crimi A, et al. Identifying the best machine learning algorithms for brain tumor segmentation, progression assessment, overall survival prediction in the brats challenge [Preprint] (2018). Available at: http://arxiv.org/1811.02629.

[15] Bakas S Akbari H Sotiras A Bilello M Rozycki M Kirby J Segmentation labels, radiomic features for the pre-operative scans of the TCGA-LGG collection [data set]. The cancer imaging archive. (2017) 286. 10.7937/K9/TCIA.2017.KLXWJJ1Q PMC5685212 28872634

[16] Amin J Sharif M Gul N Yasmin M Shad SA. Brain tumor classification based on DWT fusion of MRI sequences using convolutional neural network. Pattern Recognit Lett. (2020) 129:115–22. 10.1016/j.patrec.2019.11.016

[17] Usman K Rajpoot K. Brain tumor classification from multi-modality MRI using wavelets and machine learning. Pattern Anal Appl. (2017) 20(3):871–81. 10.1007/s10044-017-0597-8

[18] Rehman A Khan MA Saba T Mehmood Z Tariq U Ayesha N. Microscopic brain tumor detection and classification using 3D CNN and feature selection architecture. Microsc Res Tech. (2021) 84(1):133–49. 10.1002/jemt.23597 32959422

[19] Haq EU Jianjun H Li K Haq HU Zhang T. An MRI-based deep learning approach for efficient classification of brain tumors. J Ambient Intell Humaniz Comput. (2021):1–22. 10.1007/s12652-021-03535-9

[20] Shahzadi I Tang TB Meriadeau F Quyyum A. CNN-LSTM: cascaded framework for brain tumour classification. In *2018 IEEE-EMBS Conference on Biomedical Engineering and Sciences (IECBES)*. Kuching, Malaysia: IEEE (2018). p. 633–637.

[21] Mzoughi H Njeh I Wali A Slima MB BenHamida A Mhiri C Mahfoudhe KB. Deep multi-scale 3d convolutional neural network (CNN) for MRI gliomas brain tumor classification. J Digit Imaging. (2020) 33:903–15. 10.1007/s10278-020-00347-9 32440926 PMC7522155

[22] Rakelly K Shelhamer E Darrell T Efros A Levine S. Conditional networks for few-shot semantic segmentation (2018).

[23] Roth HR Yang D Xu Z Wang X Xu D. Going to extremes: weakly supervised medical image segmentation. Mach Learn Knowl Extr. (2021) 3(2):507–24. 10.3390/make3020026

[24] Kolesnikov A Lampert CH. Seed, expand and constrain: three principles for weakly-supervised image segmentation. In *European conference on computer vision*. Amsterdam, Netherlands: Springer (2016). p. 695–711.

[25] Xiao M Zhang L Shi W Liu J He W Jiang Z. A visualization method based on the grad-cam for medical image segmentation model. In *2021 International Conference on Electronic Information Engineering and Computer Science (EIECS)*. Larisa, Greece: IEEE (2021). p. 242–247.

[26] Guan S Loew M. A sneak attack on segmentation of medical images using deep neural network classifiers. In *2021 IEEE Applied Imagery Pattern Recognition Workshop (AIPR)*. Washington, DC, USA: IEEE (2021). p. 1–8.

[27] Eykholt K Evtimov I Fernandes E Li B Rahmati A Xiao C Prakash A Kohno T Song D. Robust physical-world attacks on deep learning visual classification. In *Proceedings of the IEEE Conference on Computer Vision and Pattern Recognition*. Salt Lake City, Utah: IEEE (2018). p. 1625–1634.

[28] Szegedy C Zaremba W Sutskever I Bruna J Erhan D Goodfellow I Fergus R. Intriguing properties of neural networks [Preprint] (2013). Available at: http://arxiv.org/1312.6199.

[29] Ribeiro MT Singh S Guestrin C. Why should I trust you? Explaining the predictions of any classifier. In *Proceedings of the 22nd ACM SIGKDD International Conference on Knowledge Discovery and Data Mining*. San Francisco, CA, USA: Association for Computing Machinery (2016). p. 1135–1144.

[30] Palatnik de Sousa I Maria Bernardes Rebuzzi Vellasco M Costa da Silva E. Local interpretable model-agnostic explanations for classification of lymph node metastases. Sensors. (2019) 19(13):2969. 10.3390/s19132969 31284419 PMC6651753

[31] Schallner L Rabold J Scholz O Schmid U. Effect of superpixel aggregation on explanations in lime: a case study with biological data. In *Joint European Conference on Machine Learning and Knowledge Discovery in Databases*. Springer (2019). p. 147–158.

[32] Malhi A Kampik T Pannu H Madhikermi M Främling K. Explaining machine learning-based classifications of in-vivo gastral images. In *2019 Digital Image Computing: Techniques and Applications (DICTA)*. Perth, WA, Australia: IEEE (2019). p. 1–7

[33] Petsiuk V Das A Saenko K. Rise: randomized input sampling for explanation of black-box models [Preprint] (2018). Available at: http://arxiv.org/1806.07421.

[34] Yu L Xiang W Fang J Chen Y-PP Zhu R. A novel explainable neural network for Alzheimer’s disease diagnosis. Pattern Recognit. (2022) 131:108876. 10.1016/j.patcog.2022.108876

[35] Ren X Malik J. Learning a classification model for segmentation. In *IEEE International Conference on Computer Vision*. Vol. 2. Nice, France: IEEE Computer Society (2003). p. 10–10.

[36] Ouyang C Biffi C Chen C Kart T Qiu H Rueckert D. Self-supervision with superpixels: training few-shot medical image segmentation without annotation. In *European Conference on Computer Vision*. Glasgow, UK: Springer (2020). p. 762–780.

[37] Huang Q Huang Y Luo Y Yuan F Li X. Segmentation of breast ultrasound image with semantic classification of superpixels. Med Image Anal. (2020) 61:101657. 10.1016/j.media.2020.101657 32032899

[38] Achanta R Shaji A Smith K Lucchi A Fua P Süsstrunk S. SLIC superpixels compared to state-of-the-art superpixel methods. IEEE Trans Pattern Anal Mach Intell. (2012) 34(11):2274–82. 10.1109/TPAMI.2012.120 22641706

[39] He K Zhang X Ren S Sun J. Deep residual learning for image recognition. *Corr* (2015). abs/1512.03385.

[40] Kingma DP Ba J. Adam: a method for stochastic optimization [Preprint] (2014). Available at: http://arxiv.org/1412.6980.

[41] Van der Walt S Schönberger JL Nunez-Iglesias J Boulogne F Warner JD Yager N Gouillart E Yu T. scikit-image: image processing in python. PeerJ. (2014) 2:e453. 10.7717/peerj.453 25024921 PMC4081273

[42] Ronneberger O Fischer P Brox T. U-net: convolutional networks for biomedical image segmentation. In *International Conference on Medical Image Computing and Computer-Assisted Intervention*. Munich, Germany: Springer (2015). p. 234–241.

[43] Selvaraju R Cogswell M Das A Vedantam R Parikh D Batra D. Grad-CAM: visual explanations from deep networks via gradient-based localization. arxiv. *Preprint posted online*, 7 (2016).

